# Persistent white matter vulnerability in a mouse model of mild traumatic brain injury

**DOI:** 10.1186/s12868-022-00730-y

**Published:** 2022-07-18

**Authors:** Prashanth S. Velayudhan, Jordan J. Mak, Lisa M. Gazdzinski, Anne L. Wheeler

**Affiliations:** 1 Program in Neurosciences and Mental Health, The Hospital for Sick Children, Toronto, ON M5G 0A4 Canada; 2 Department of Physiology, University of Toronto, Toronto, ON M5S 1A8 Canada

**Keywords:** Mild traumatic brain injury, Window of vulnerability, White matter, Mouse model, Sex differences, Corpus callosum, Optic tract, Silver stain, Y-maze test, Visual cliff test

## Abstract

**Background:**

Following one mild traumatic brain injury (mTBI), there is a window of vulnerability during which subsequent mTBIs can cause substantially exacerbated impairments. Currently, there are no known methods to monitor, shorten or mitigate this window.

**Methods:**

To characterize a preclinical model of this window of vulnerability, we first gave male and female mice one or two high-depth or low-depth mTBIs separated by 1, 7, or 14 days. We assessed brain white matter integrity using silver staining within the corpus callosum and optic tracts, as well as behavioural performance on the Y-maze test and visual cliff test.

**Results:**

The injuries resulted in windows of white matter vulnerability longer than 2 weeks but produced no behavioural impairments. Notably, this window duration is substantially longer than those reported in any previous preclinical vulnerability study, despite our injury model likely being milder than the ones used in those studies. We also found that sex and impact depth differentially influenced white matter integrity in different white matter regions.

**Conclusions:**

These results suggest that the experimental window of vulnerability following mTBI may be longer than previously reported. Additionally, this work highlights the value of including white matter damage, sex, and replicable injury models for the study of post-mTBI vulnerability and establishes important groundwork for the investigation of potential vulnerability mechanisms, biomarkers, and therapies.

**Supplementary Information:**

The online version contains supplementary material available at 10.1186/s12868-022-00730-y.

## Introduction

After sustaining a mild traumatic brain injury (mTBI), patients enter a window of vulnerability during which a subsequent injury can result in longer lasting and more severe symptoms. In previous preclinical studies examining the window of vulnerability, rodents have been given two mTBIs separated by a set of intervals with the outcome of interest being which of those intervals resulted in exacerbated damage relative to a single mTBI [1–5]. Using a variety of injury models and assessment methodologies, those studies reported windows of vulnerability ranging between 5 days and 2 weeks. However, they share a few limitations which have impeded the translational relevance of the findings.

To date, no preclinical vulnerability study has included female subjects, which are known to experience different outcomes than males following mTBI [6, 7]. Though clinical studies tend to report worse outcomes in females and preclinical studies tend to report worse outcomes in males, reporting on post-mTBI sex differences is generally mixed [6], making the inclusion of both sexes an essential component of any well-characterized vulnerability model.

Previous studies have also not used white matter integrity as a measure of damage when defining vulnerability, despite this measure being increasingly recognized as one of the most sensitive measures of damage and strongest correlates of impairment following mTBI [8–10].

Finally, previous studies on post-mTBI vulnerability have not examined how changes in injury parameters influence outcomes, making it challenging to integrate findings across different sets of experimental conditions.

In this study, we aimed to characterize a mouse model of the window of vulnerability that uses white matter integrity as a primary outcome and accounts for more than one sex and set of injury parameters.

## Materials and methods

### Animals and experimental design

Male and female C57BL6/J mice were randomly assigned to one of three injury groups (two mTBIs, one sham operation followed by one mTBI, or two sham operations), to one of two impact depths (low-depth mTBI or high-depth mTBI), and to one of three inter-operation intervals (1 day, 1 week, or 2 weeks) using an online random number generator. Mice receiving one sham operation followed by one mTBI were included to identify inter-operation intervals for which two mTBIs resulted in worse outcomes than one mTBI. Mice receiving two sham operations were included to obtain baseline measures for the included outcome assessment methods. The experimental unit of all analyses was a single mouse. All mice were 8 weeks old at the time of their first operation. A total of 144 mice were used in this study (N = 4 mice/sex/impact depth/inter-operation intervals/injury groups; sample sizes based on previous pilot data). All 144 mice were included for behavioural analysis. A small subset of double-sham-receiving mice were included for histological analysis to serve as negative controls. Some mice receiving one or two mTBIs were excluded from histological analysis due to tissue damage. Final sample sizes are included in Additional file 1: Table S1. A priori exclusion criteria included mice that showed signs of skull fracture during the mTBI procedure or during euthanasia, but this criterion was not met by any subjects. For each inter-injury interval and impact depth group, operations were delivered in cycling order across sex and injury group combinations to avoid operation time as a potential confounder.

### mTBI procedure

Mice were given subcutaneous injections of sustained-release buprenorphine (0.100 mL at 0.600 mg/mL), bupivacaine (0.05 mL at 0.25%), xylocaine (0.05 mL at 0.2%), and Lactated Ringer’s with 5% dextrose (0.75 mL) for analgesic support and hydration. Following induction of anesthesia with isoflurane (4% for induction and 2.0–2.5% for maintenance), the hair was removed from the scalp using depilatory cream and the mice were positioned in a stereotactic frame with their heads fixed using zygomatic cuffs (Stoelting Co., USA). An electromagnetic controlled cortical impactor (ImpactOne, Leica Biosystems, Canada) with a 5 mm metal tip was used to deliver an mTBI to mice at bregma with a speed of 2.0 m/s, a dwell time of 200 ms, and a depth of 1.2 mm or 1.6 mm beyond the scalp surface. Mice that experienced over 5 s of apnea received chest compressions until regular breathing resumed to minimize brain damage by hypoxic injury. The mice were given approximately 30 min of recovery time on a heating pad before being returned to home cages.

### Behavioural assessment

The experimenter was blinded to mouse group allocation during all behavioural testing. One week after their second injury, mice were assessed on the Y-maze to assess short-term spatial memory. Mice were given 10 min to freely roam in a Y-shaped maze containing three equally sized arms while their positions were tracked by EthoVision XT automated video tracking software (Noldus, Wageningen, the Netherlands). The spontaneous alternations index was calculated as the percentage of successful alternations (visits to three different arms in succession) out of maximum possible alternations (visits to three non-unique arms in succession). Motivation for this variant of the Y-maze test has been demonstrated to be driven by an innate preference in the mice for exploration, and does not require the usage of external motivators or pre-testing habituation to be effective [11]. Mice were then assessed on the visual cliff test to assess visual system integrity. In this test, mice were given 5 min to freely roam a square chamber containing a clear plexiglass floor that was elevated 1.0 m off the ground. One half of the plexiglass floor was covered with an opaque checkered sticker to generate the visual effect of a steep cliff at the sticker’s border. The time spent on the safe, checkered side of the chamber was manually recorded.

### Euthanasia and perfusion fixation

Two days after behavioural testing, mice were anesthetized to unconsciousness with an intraperitoneal injection of ketamine (150 mg/kg) and xylazine (10 mg/kg) before being euthanized via transcardial perfusion with 30 mL of heparinized phosphate buffer solution (PBS; 1 USP unit heparin/mL PBS) followed by 30 mL of 4% paraformaldehyde in PBS at a rate of 1 mL/min. Following the perfusion, brains were dissected out of the skulls and immersed in 4% paraformaldehyde in PBS for 1 day, then stored in PBS with 0.02% sodium azide until further processing. This method of euthanasia and perfusion fixation has been effectively used by our lab for tissue collection and staining previously [12].

### Tissue collection and silver staining

Brains were cryoprotected for cryostat sectioning by soaking in 15% sucrose in PBS for 1 day followed by 30% sucrose in PBS with 0.02% sodium azide for at least 2 days. 40 μm frozen sections were collected from each of the following locations: 100 µm anterior to bregma, 100 µm posterior to bregma, 1500 µm posterior to bregma, and 1700 µm posterior to bregma. Sections were silver stained to assess white matter integrity using the FD NeuroSilver Kit II (FD NeuroTechnologies Inc., USA). One image was acquired at 20× magnification on a Nikon Eclipse Ni-U microscope from the center of the corpus callosum from each of the two anterior sections and from the left or right optic tract from each of the two posterior sections.

Silver staining was quantified using Fiji [13] by an observer blinded to sample identity and group (PV). Images were duplicated and placed side-by-side to facilitate visual comparison. One copy of the stained image was converted to 8-bit type and an area threshold was manually adjusted to best match the area of silver staining present in the unaltered image. The percentage area of staining above the applied threshold within the region of interest (either the corpus callosum or optic tract) was recorded as the primary outcome of the study. A subset (25%) of all staining measures was quantified by a second blinded observer (JM) to calculate the intraclass correlation coefficient (ICC) as a metric of inter-rater reliability. Mean staining values averaged over two corpus callosum or optic tract quantifications per mouse were used for statistical analysis.

### Statistical analyses

To evaluate behavioural impairments associated with the injury model, two-tailed, two sample Welch’s t-tests were used to compare mice receiving 1 mTBI or 2 mTBIs with shams for each inter-injury interval.

To identify vulnerable inter-injury intervals defined by silver staining, one-tailed, two sample Welch’s t-tests were used to compare mice receiving 1 mTBI with mice receiving 2 mTBIs for each inter-injury interval and white matter region. Intervals for which mice receiving 2 mTBIs had significantly higher silver staining were considered vulnerable.

To identify the effects of sex and impact depth on white matter integrity, a multiple linear regression model was evaluated in which the dependent variable was silver staining area and the explanatory variables were number of injuries, inter-injury interval, sex, and impact depth for each white matter region.

## Results

### Injuries following low-depth and high-depth impacts were mild in nature

Mice receiving 1.2 mm-depth mTBIs experienced no apnea while mice receiving 1.6 mm-depth mTBIs experienced 41 ± 33 (mean ± standard deviation) seconds of apnea. Impacts resulted in no skull fractures or visible brain contusion.

### No impairments on Y-maze test or visual cliff test following one or two mTBIs

No significant differences were found between mice receiving one or two mTBIs with mice receiving sham operations for any inter-injury interval in the Y-maze test or in the visual cliff test (summary statistics presented in Additional file 1: Table S2). This result held when sex and impact depth groups were examined independently. This observation emphasizes the mild nature of this model and suggests that visual perception and short-term spatial memory were minimally affected following 1 or 2 mTBIs.

### Increased white matter vulnerability to repeat mTBI for at least 2 weeks post-injury

**Fig. 1:**
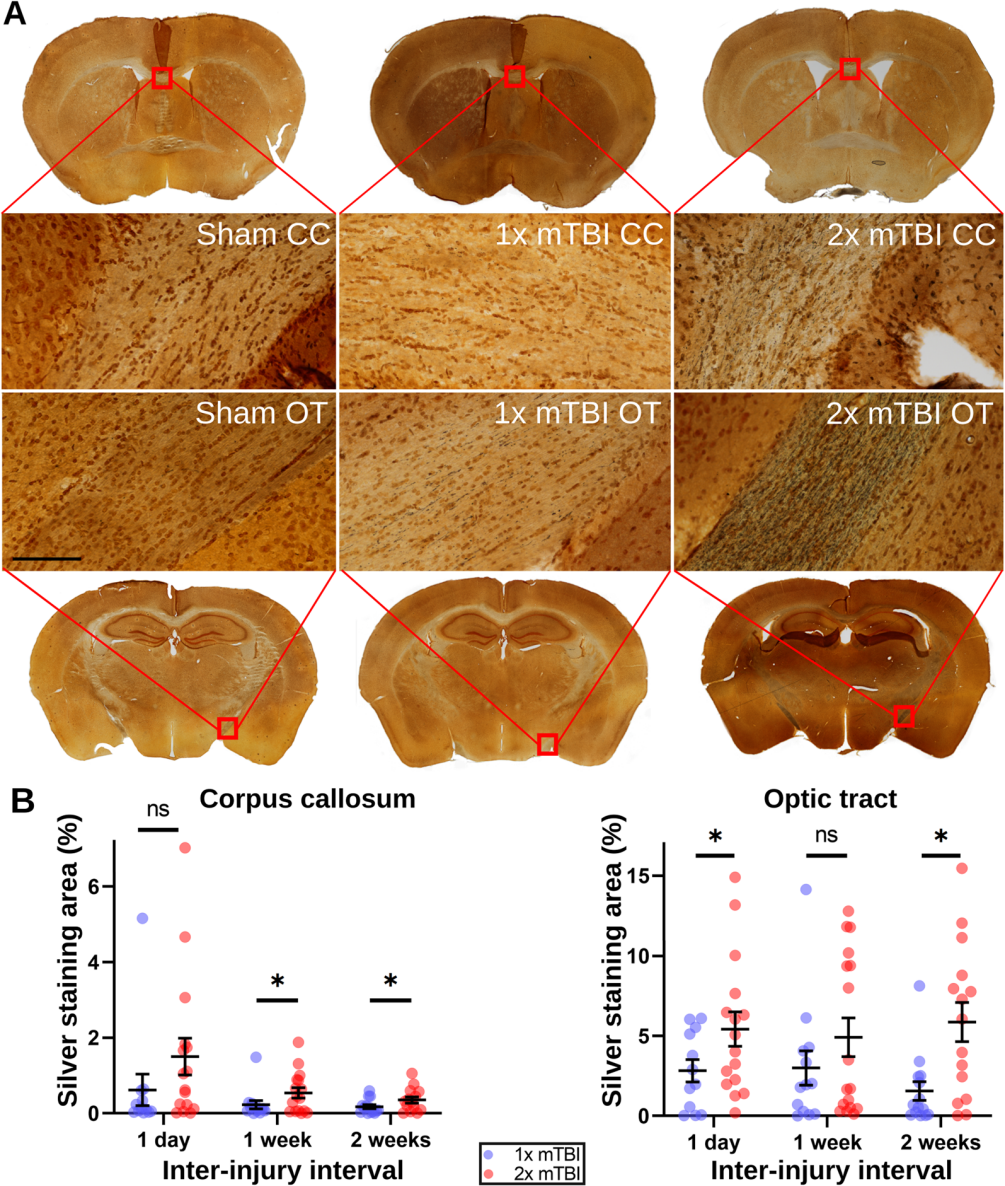
Silver staining in the corpus callosum and optic tracts. **A** Representative images of silver staining in mice receiving two sham operations, one sham operation and one mTBI, or two mTBIs in the corpus callosum (CC; top) and optic tract (OT; bottom). Scale bar represents 10 μm. **B** Quantified silver staining % area. * = p < 0.05. All data are presented as the mean ± the standard error of the mean. Sample sizes (1D = 1 day, 1X = 1 × mTBI, 2X = 2 × mTBI): CC-1D-1X: 12; CC-1D-2X: 16; CC-1 W-1X: 13; CC-1 W-2X: 16; CC-2 W-1X: 14; CC-2 W-2X: 14; OT-1D-1X: 13; OT-1D-2X: 15; OT-1 W-1X: 13; OT-1 W-2X: 15; OT-2 W-1X: 14; OT-2 W-2X: 15. Note that the extent of counter-staining (brownish-orange background) present on the tissue slices is an artifact of processing the tissue with the silver staining kit and is not related to the extent of silver deposited on the tissue itself (black specks and lines)

Percentage silver staining area was used to quantify the extent of white matter damage in the optic tract and corpus callosum regions of mice. The ICC agreement between independent quantifiers of silver staining was found to be 0.604 (considered ‘good’ [14]). Figure 1 shows the amount of silver staining in the corpus callosum and optic tracts of mice receiving two sham operations, one sham operation followed by one mTBI, or two mTBIs, with inter-operation intervals of 1 day, 1 week, or 2 weeks. Increased silver staining in mice receiving two mTBIs compared to mice receiving one mTBI was observed in the corpus callosum of mice receiving operations separated by 1 week (p_2x–1x_ = 0.041) or 2 weeks (p_2x–1x_ = 0.038), as well as in the optic tracts of mice receiving operations separated by 1 day (p_2x–1x_ = 0.026) or 2 weeks (p_2x–1x_ = 0.0024), but not in the corpus callosum of mice receiving operations separated by 1 day (p_2x–1x_ = 0.091) or in the optic tracts of mice receiving operations separated by 1 week (p_2x–1x_ = 0.071). P-values are listed in Additional file 1: Table S3. The lack of a significant difference observed in the latter two conditions appears to be driven by individual outliers in the single mTBI groups. These findings demonstrate that the post-mTBI window of vulnerability associated with this injury model, defined as the duration following one mTBI for which a second identical mTBI results in exacerbated white matter damage, is longer than 2 weeks.

### Sex and impact depth differentially affect white matter integrity in the corpus callosum and optic tracts

**Fig. 2:**
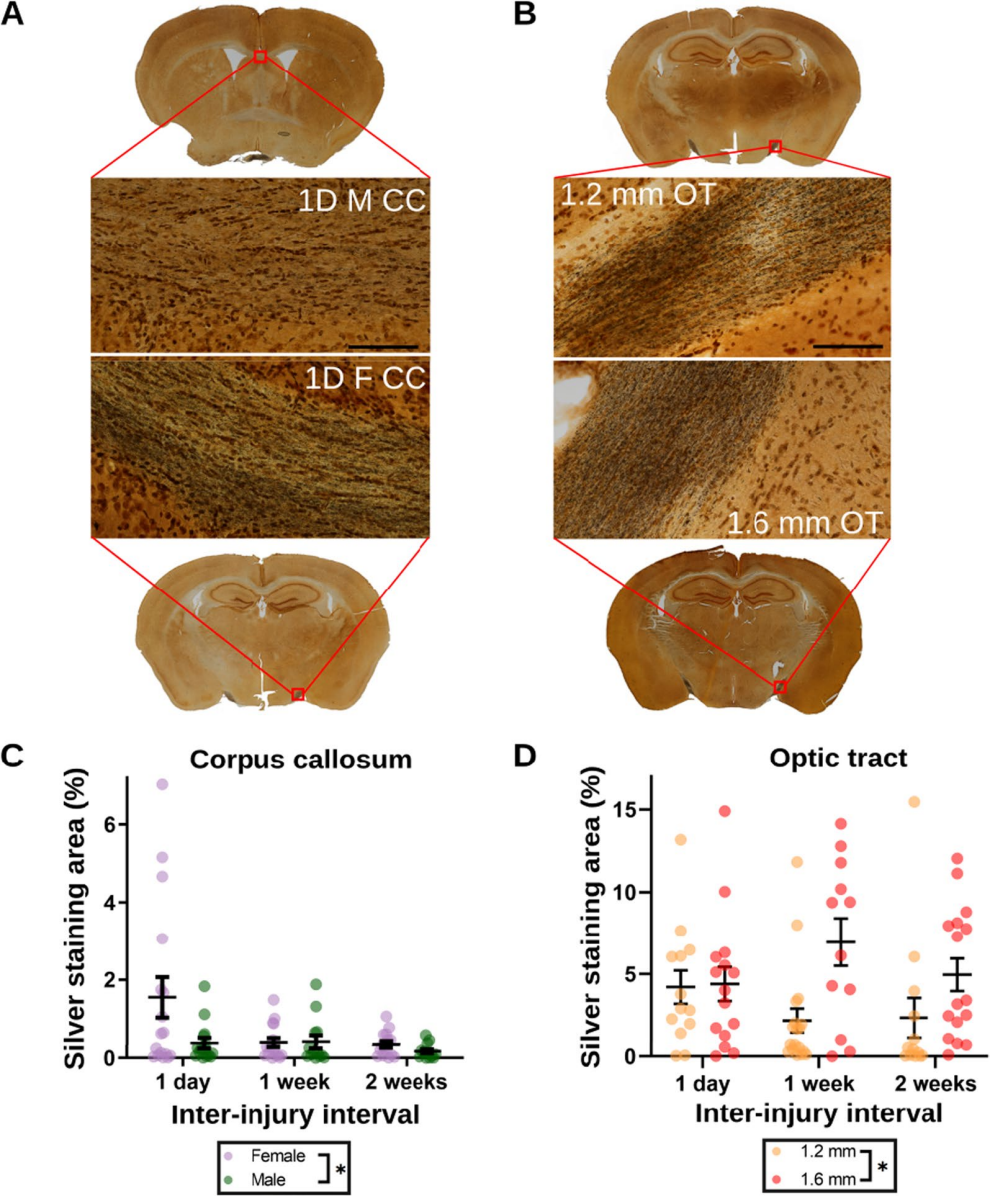
Silver staining outcomes stratified by sex in the corpus callosum or by impact depth in the optic tract. **A**–**B** Representative silver staining images in the corpus callosum **A** and optic tract **B**. **C**–**D** Quantified silver staining % area in the corpus callosum **C** and optic tract **D**. * = p < 0.05. Sample sizes (1D = 1 day, 1x = 1 × mTBI, 2x = 2 × mTBI, 1.2 = 1.2 mm depth, 1.6 = 1.6 mm depth): CC-1D-F: 15; CC-1D-M: 13; CC-1 W-F: 16; CC-1 W-M: 13; CC-2 W-F: 15; CC-2 W-M: 13; OT-1D-1.2: 13; OT-1D-1.6: 15; OT-1 W-1.2: 16; OT-1 W-1.6: 12; OT-2 W-1.2: 13; OT-2 W-1.6: 16. Note that the extent of counter-staining (brownish-orange background) present on the tissue slices is an artifact of processing the tissue with the silver staining kit and is not related to the extent of silver deposited on the tissue itself (black specks and lines)

Multiple linear regression was used to determine if sex or impact depth influenced white matter damage in the optic tract or corpus callosum following mTBI. Females had significantly more silver staining than males in the corpus callosum (β = 0.49, p = 0.032; Fig. 2A) and 1.6 mm-depth impacts resulted in significantly more silver staining than 1.2 mm-depth impacts in the optic tract (β = 2.5, p = 0.0026; Fig. 2B). P-values of other terms are provided in Additional file 1: Tables S4, S5. Intriguingly, these results suggest that the corpus callosum and optic tract regions differ in their sensitivity to the parameters of sex and impact depth.

## Discussion

To our knowledge, this is the first study on post-mTBI vulnerability which examined both male and female subjects. It is also the first study on post-mTBI vulnerability to use white matter integrity as a primary measure of brain damage or to examine more than one set of injury parameters.

Cognitive impairments are frequently observed in mouse models of multiple mTBI. In Schwab et al. [15], researchers gave mice 3 mTBIs separated 1 day apart and found impairments in spatial learning and memory 1 week later, as evidenced by the Morris Water Maze (MWM) test. Also using the MWM test, Yang et al. [16] found impaired spatial learning in mice that received 4 mTBIs separated across 10 days at 15 days after their last injury. A study by Feng et al. [17] found that for at least 4 weeks following 6 mTBIs, mice demonstrated impaired short-term memory in the novel object recognition test and impaired fear learning in the fear conditioning test.

Despite the tendency for mouse models of repeated mTBI to result in well-defined cognitive impairments, there are a number of reports which have found no detectable changes in mouse behaviour or cognition. For example, in Huh et al. [18], researchers observed pathological alterations in ventricle and white matter structure following two mTBIs to young rat pups, but no impairments on the Morris Water Maze test. Another example of a study which found pathological alterations but no impairments in cognition specifically is Semple et al. [19]. In this study, researchers found that 2 mTBIs did not result in any impairments in learning or memory on the novel object recognition test or radial arm water maze test.

Whether an rmTBI will result in cognitive impairments likely depends on a combination of test sensitivity and timing, animal physiology, and the details of the injury model used and number of injuries delivered. Injury paradigms such as the one employed in this study may be reflective of mTBIs or concussions which do not result in serious symptoms on their own, but may prime the brain to sustain sufficient pathological alterations on a subsequent mTBI for neurological impairments to manifest.

The white matter-defined window of vulnerability associated with this injury model appeared to be longer than reported in any prior study of post-mTBI vulnerability. Though performance on the Y-maze and visual cliff tests were not impacted by the mTBIs delivered in this study, the compound white matter damage observed between mice receiving two mTBIs compared to mice receiving one mTBI suggest that additional injuries of the same severity would eventually result in sufficient white matter damage to produce observable behavioural impairments.

We did not explicitly calculate effect sizes associated with the differences between one and two mTBIs, as we interpreted any exacerbation of injury caused by a second mTBI within a given interval would eventually extrapolate to clinically significant impairment following repeated mTBIs. As white matter integrity did not return to baseline levels for any inter-injury interval (and did not trend towards returning to baseline levels in the case of the optic tract), it is possible that the window of vulnerability associated with our model may never close. This may analogously suggest that mTBIs can result in some degree of irreversibly increased vulnerability to subsequent injuries.

In Longhi et al. [1], mTBIs were delivered by a piston with a silicone tip striking 3 mm past the surface of the mouse’s skull, fixed in position, with a velocity of 5.0 m/s. The most sensitive outcome measure was the Morris Water Maze test, which identified an associated window of vulnerability that was shorter than one week. In a two-part study by Vagnozzi and Tavazzi et al. from 2007 [2, 3], mTBIs were delivered by dropping a 450 g weight from a height of 1 m onto the free-moving heads of rats. Several outcome measures, including a variety of biochemical indices of oxidative stress, nitrosative stress, and mitochondrial metabolites, collectively identified a window of vulnerability of approximately 5 days. In Prins et al. [4], mTBIs were delivered by a piston displacing the free-moving heads of mice by 8 mm at 36 psi. Both ^14^C-2-deoxy-d-glucose autoradiography and the novel object recognition test revealed an associated window of vulnerability that was shorter than 5 days. Finally, in Selwyn et al. [5], mTBIs were delivered by a pulse of water directly impacting the brains of rats with a force of 1.14 ± 0.5 atm. The most sensitive outcome measure was [^18^F]-fluorodeoxyglucose uptake measured by positron emission tomography (FDG-PET), which identified an associated window of vulnerability that was shorter than 15 days.

In contrast, the reported window duration in this study was greater than 2 weeks and the injuries consisted of a piston with a metal tip striking 1.2–1.6 mm past the surface of the mouse’s intact skull with a velocity of 2.0 m/s. Though it is not possible to precisely infer relative impact severities across injury models with certainty, comparison of the injury parameters suggests that this injury model is among the mildest, if not the mildest, of the models used in post-mTBI vulnerability studies to date. We suspect that the apparent discrepancy between the mildness of this injury model and the length of its associated window of vulnerability is the result of our primary outcome measure, white matter integrity, being a substantially more sensitive measure of post-mTBI damage than the metrics used by those previously mentioned studies. Alternative explanations for the discrepancy may be that this injury model was effectively more severe than the previously mentioned ones or that this study had a greater statistical power to detect differences between one and two mTBIs than those previous studies. An important consideration is that the relative sensitivity of various methods may vary across injury model parameters.

We observed that females showed more white matter damage than males, which does not match the majority of sex effects reported in experimental mTBI, but does agree with the majority of sex effects reported in clinical studies [6]. Sex differences in white matter integrity are not commonly reported in preclinical mTBI literature. One example finding is in Wright et al. [19], in which male rats were found to have reduced myelin basic protein (MBP) expression in the corpus callosum than female rats following mTBI. The greater white matter damage observed in the corpus callosum of females in this study may be explained by different white matter regions being differentially sensitive to mTBI, rather than all white matter structures being more vulnerable to mTBI for one of the sexes. The pattern of structure-specific sex differences in white matter damage following mTBI is being increasingly reported in the clinical setting [20–22].

We also observed that impact depth was a significant predictor for white matter damage in the optic tract, but not in the corpus callosum. An additional observation noticeable in Fig. 1 and supported by the inter-injury interval effects in the multivariate results (Additional file 1: Tables S3, S4), is that the difference in silver staining outcome appears to decrease with increasing inter-injury interval for the corpus callosum but not for the optic tract, suggesting that the window of vulnerability may resolve more slowly in the optic tract region. Future replications in other models will be required to determine if persistent white matter vulnerability in the optic tract is a specific feature of experimental injury models similar to the one used in this study.

Limitations of this study include not determining the full duration of the window of vulnerability associated with the injury model, only examining one metric of white matter damage, not controlling for estrus stage in female mice, and potential confounds across sexes or inter-injury intervals due to differences in analgesia uptake and biophysical properties of the skull. Additionally, although sex and impact depth parameters were observed to affect white matter integrity, the experiment was not powered to answer whether they differentially affect the window of vulnerability. As well, we did not include multiple comparisons to control for increases in the familywise error rate across the reported analyses. We consider this work to be preliminary in nature and expect future replications and extensions of this work to further validate the findings reported here.

## Conclusions

In this study, we found that for a relatively mild model of mTBI, the associated window of vulnerability was greater than 2 weeks, which is longer than reported in any previous preclinical study of post-mTBI vulnerability. We also found that female mice had increased white matter damage in the corpus callosum following mTBI relative to male mice, and that mice receiving 1.6 mm-depth impacts exhibited more white matter damage in the optic tracts relative to mice receiving 1.2 mm-depth impacts. As no behavioural impairment was detected, behavioural assessments are deemed to be less sensitive measures for characterizing the duration of the window of vulnerability. Collectively, these findings promote silver staining of white matter damage as a highly sensitive measure of post-mTBI damage and suggest that previous studies may have underestimated the duration of vulnerability following their models of experimental mTBI. Additionally, sex and impact depth have been identified as important factors for the degree of vulnerability following mTBI, demonstrating the importance of their consideration for future studies on this subject. These results serve as foundational work towards better understanding the mechanisms of the post-mTBI window of vulnerability. This window is especially concerning for populations that are highly prone to mTBIs, such as children, military personnel, the elderly, athletes, victims of domestic abuse, and sex workers.

## Supplementary Information

**Additional file 1: Table S1.** Sample sizes for all experimental groups. A small subset of double-sham-receiving mice were included for histological analysis to serve as negative controls. Some mice receiving one or two mTBIs were excluded from histological analysis due to tissue damage. **Table S2.** Summary statistics and two-tailed t-test p-values of comparisons between mice receiving 1 or 2 mTBIs against mice receiving sham operations on the spontaneous alternations index and maximum number of alternations (total arm entrances—2) of the Y-maze test and the proportion of time spent on the safe side of the visual cliff test. **Table S3.** Summary statistics and one-tailed t-test p-values of comparisons of silver staining area in the corpus callosum or optic tract regions between mice receiving 1 mTBI and mice receiving 2 mTBIs. * = p< 0.05, ** = p< 0.01. **Table S4.** Output of multiple linear regression examining the effects of sex and impact severity on white matter damage in the corpus callosum. * = p< 0.05, ** = p< 0.01, *** = p< 0.001. **Table S5.** Output of multiple linear regression examining the effects of sex and impact severity on white matter damage in the optic tract. * = p< 0.05, ** = p< 0.01, *** = p< 0.001.

## Data Availability

All data generated or analysed during this study are available from the corresponding author upon reasonable request.

## Abbreviations

β-APP: β-Amyloid precursor protein
CC: Corpus callosum
FDG-PET: Fluorodeoxyglucose positron emission tomography
GFAP: Glial fibrillary acidic protein
Iba1: Ionized calcium binding adaptor molecule 1
ICC: Intraclass correlation coefficient
MAP2: Microtubule-associated protein 2
mTBI: Mild traumatic brain injury
OT: Optic tract
PBS: Phosphate buffered saline
