# Supplementary material for: Persistent white matter vulnerability in a mouse model of mild traumatic brain injury
Source: BMC Neurosci. 2022 Jul 18;23:46. doi: 10.1186/s12868-022-00730-y (PMC9290236; doi:10.1186/s12868-022-00730-y)
Supplement: Supplementary file 1 — Additional file 1: Table S1. Sample sizes for all experimental groups. A small subset of double-sham-receiving mice were included for histological analysis to serve as negative controls. Some mice receiving one or two mTBIs were excluded from histological analysis due to tissue damage. Table S2. Summary statistics and two-tailed t-test p-values of comparisons between mice receiving 1 or 2 mTBIs against mice receiving sham operations on the spontaneous alternations index and maximum number of alternations (total arm entrances—2) of the Y-maze test and the proportion of time spent on the safe side of the visual cliff test. Table S3. Summary statistics and one-tailed t-test p-values of comparisons of silver staining area in the corpus callosum or optic tract regions between mice receiving 1 mTBI and mice receiving 2 mTBIs. * = p< 0.05, ** = p< 0.01. Table S4. Output of multiple linear regression examining the effects of sex and impact severity on white matter damage in the corpus callosum. * = p< 0.05, ** = p< 0.01, *** = p< 0.001. Table S5. Output of multiple linear regression examining the effects of sex and impact severity on white matter damage in the optic tract. * = p< 0.05, ** = p< 0.01, *** = p< 0.001. [file 12868_2022_730_MOESM1_ESM.docx]

**Additional file: Table S1**: Sample sizes for all experimental groups. A small subset of double-sham-receiving mice were included for histological analysis to serve as negative controls. Some mice receiving one or two mTBIs were excluded from histological analysis due to tissue damage.

| Injury group | Injury interval | Sex | Impact depth | Brain region | Sample size |
| --- | --- | --- | --- | --- | --- |
| Sham | 1 day | Male | 1.2 mm | CC | 3 |
| 1x | 1 day | Male | 1.2 mm | CC | 3 |
| 2x | 1 day | Male | 1.2 mm | CC | 3 |
| Sham | 1 day | Male | 1.2 mm | OT | 3 |
| 1x | 1 day | Male | 1.2 mm | OT | 3 |
| 2x | 1 day | Male | 1.2 mm | OT | 3 |
| Sham | 1 day | Male | 1.6 mm | CC | 1 |
| 1x | 1 day | Male | 1.6 mm | CC | 3 |
| 2x | 1 day | Male | 1.6 mm | CC | 4 |
| Sham | 1 day | Male | 1.6 mm | OT | 1 |
| 1x | 1 day | Male | 1.6 mm | OT | 3 |
| 2x | 1 day | Male | 1.6 mm | OT | 4 |
| Sham | 1 day | Female | 1.2 mm | CC | 3 |
| 1x | 1 day | Female | 1.2 mm | CC | 2 |
| 2x | 1 day | Female | 1.2 mm | CC | 5 |
| Sham | 1 day | Female | 1.2 mm | OT | 3 |
| 1x | 1 day | Female | 1.2 mm | OT | 2 |
| 2x | 1 day | Female | 1.2 mm | OT | 5 |
| Sham | 1 day | Female | 1.6 mm | CC | 1 |
| 1x | 1 day | Female | 1.6 mm | CC | 4 |
| 2x | 1 day | Female | 1.6 mm | CC | 4 |
| Sham | 1 day | Female | 1.6 mm | OT | 1 |
| 1x | 1 day | Female | 1.6 mm | OT | 4 |
| 2x | 1 day | Female | 1.6 mm | OT | 4 |
| Sham | 1 week | Male | 1.2 mm | CC | 2 |
| 1x | 1 week | Male | 1.2 mm | CC | 4 |
| 2x | 1 week | Male | 1.2 mm | CC | 4 |
| Sham | 1 week | Male | 1.2 mm | OT | 2 |
| 1x | 1 week | Male | 1.2 mm | OT | 4 |
| 2x | 1 week | Male | 1.2 mm | OT | 4 |
| Sham | 1 week | Male | 1.6 mm | CC | 0 |
| 1x | 1 week | Male | 1.6 mm | CC | 1 |
| 2x | 1 week | Male | 1.6 mm | CC | 4 |
| Sham | 1 week | Male | 1.6 mm | OT | 0 |
| 1x | 1 week | Male | 1.6 mm | OT | 1 |
| 2x | 1 week | Male | 1.6 mm | OT | 3 |
| Sham | 1 week | Female | 1.2 mm | CC | 2 |
| 1x | 1 week | Female | 1.2 mm | CC | 4 |
| 2x | 1 week | Female | 1.2 mm | CC | 4 |
| Sham | 1 week | Female | 1.2 mm | OT | 2 |
| 1x | 1 week | Female | 1.2 mm | OT | 4 |
| 2x | 1 week | Female | 1.2 mm | OT | 4 |
| Sham | 1 week | Female | 1.6 mm | CC | 2 |
| 1x | 1 week | Female | 1.6 mm | CC | 4 |
| 2x | 1 week | Female | 1.6 mm | CC | 4 |
| Sham | 1 week | Female | 1.6 mm | OT | 2 |
| 1x | 1 week | Female | 1.6 mm | OT | 4 |
| 2x | 1 week | Female | 1.6 mm | OT | 4 |
| Sham | 2 weeks | Male | 1.2 mm | CC | 2 |
| 1x | 2 weeks | Male | 1.2 mm | CC | 4 |
| 2x | 2 weeks | Male | 1.2 mm | CC | 4 |
| Sham | 2 weeks | Male | 1.2 mm | OT | 2 |
| 1x | 2 weeks | Male | 1.2 mm | OT | 4 |
| 2x | 2 weeks | Male | 1.2 mm | OT | 4 |
| Sham | 2 weeks | Male | 1.6 mm | CC | 1 |
| 1x | 2 weeks | Male | 1.6 mm | CC | 4 |
| 2x | 2 weeks | Male | 1.6 mm | CC | 4 |
| Sham | 2 weeks | Male | 1.6 mm | OT | 1 |
| 1x | 2 weeks | Male | 1.6 mm | OT | 4 |
| 2x | 2 weeks | Male | 1.6 mm | OT | 4 |
| Sham | 2 weeks | Female | 1.2 mm | CC | 3 |
| 1x | 2 weeks | Female | 1.2 mm | CC | 3 |
| 2x | 2 weeks | Female | 1.2 mm | CC | 4 |
| Sham | 2 weeks | Female | 1.2 mm | OT | 3 |
| 1x | 2 weeks | Female | 1.2 mm | OT | 3 |
| 2x | 2 weeks | Female | 1.2 mm | OT | 4 |
| Sham | 2 weeks | Female | 1.6 mm | CC | 0 |
| 1x | 2 weeks | Female | 1.6 mm | CC | 4 |
| 2x | 2 weeks | Female | 1.6 mm | CC | 4 |
| Sham | 2 weeks | Female | 1.6 mm | OT | 0 |
| 1x | 2 weeks | Female | 1.6 mm | OT | 4 |
| 2x | 2 weeks | Female | 1.6 mm | OT | 4 |

**Additional file: Table S2**: Summary statistics and two-tailed t-test p-values of comparisons between mice receiving 1 or 2 mTBIs against mice receiving sham operations on the spontaneous alternations index and maximum number of alternations (total arm entrances – 2) of the Y-maze test and the proportion of time spent on the safe side of the visual cliff test.

| Interval | Sham mean ± SD (%) | 1x mTBI mean ± SD (%) | p-value | 2x mTBI mean ± SD (%) | p-value |
| --- | --- | --- | --- | --- | --- |
| Y-maze test (spontaneous alternations index) | | | | | |
| 1 day | 46.4 ± 12.9 | 42.3 ± 10.0 | 0.315 | 47.4 ± 11.0 | 0.817 |
| 1 week | 46.5 ± 12.7 | 49.2 ± 8.0 | 0.470 | 52.4 ± 8.6 | 0.139 |
| 2 weeks | 47.9 ± 8.4 | 46.9 ± 10.1 | 0.622 | 49.7 ± 11.3 | 0.755 |
| Y-maze test (maximum alternations) | | | | | |
| 1 day | 31.7 ± 10.2 | 33.8 ± 5.1 | 0.462 | 31.0 ± 8.0 | 0.833 |
| 1 week | 31.3 ± 8.1 | 27.1 ± 7.8 | 0.152 | 29.9 ± 9.9 | 0.673 |
| 2 weeks | 30.1 ± 8.2 | 30.4 ± 6.4 | 0.905 | 33.2 ± 10.7 | 0.361 |
| Visual cliff test | | | | | |
| 1 day | 56.2 ± 12.8 | 53.1 ± 11.8 | 0.477 | 58.8 ± 13.3 | 0.584 |
| 1 week | 58.8 ± 12.7 | 54.7 ± 19.4 | 0.493 | 56.4 ± 12.8 | 0.599 |
| 2 weeks | 52.3 ± 14.5 | 59.1 ± 11.6 | 0.153 | 52.8 ± 8.1 | 0.905 |

**Additional file: Table S3**: Summary statistics and one-tailed t-test p-values of comparisons of silver staining area in the corpus callosum or optic tract regions between mice receiving 1 mTBI and mice receiving 2 mTBIs. * = p < 0.05, ** = p < 0.01.

| Interval | 1x mTBI mean ± SD (%) | 2x mTBI mean ± SD (%) | p-value |
| --- | --- | --- | --- |
| Corpus callosum | | | |
| 1 day | 0.620 ± 1.45 | 1.50 ± 1.95 | 0.0907 |
| 1 week | 0.228 ± 0.387 | 0.541 ± 0.545 | 0.0413* |
| 2 weeks | 0.176 ± 0.190 | 0.356 ± 0.310 | 0.0384* |
| Optic tract | | | |
| 1 day | 2.82 ± 2.43 | 5.43 ± 4.28 | 0.0264* |
| 1 week | 2.99 ± 3.86 | 5.52 ± 4.98 | 0.0715 |
| 2 weeks | 1.55 ± 2.19 | 5.87 ± 4.75 | 0.00239** |

**Additional file: Table S4**: Output of multiple linear regression examining the effects of sex and impact severity on white matter damage in the corpus callosum. * = p < 0.05, ** = p < 0.01, *** = p < 0.001.

| Coefficient | β estimate | Standard error | t value | Pr(>\|t\|) |
| --- | --- | --- | --- | --- |
| Intercept | 1.11 | 0.284 | 3.91 | 0.000193*** |
| Number of injuries = 2x (reference: 1x) | 0.451 | 0.226 | 2.00 | 0.0488* |
| Inter injury interval = 1 week (reference: 1 day) | -0.724 | 0.275 | -2.63 | 0.0102* |
| Inter injury interval = 2 weeks (reference: 1 day) | -0.824 | 0.277 | -2.97 | 0.00394** |
| Sex = female (reference: male) | 0.493 | 0.225 | 2.19 | 0.0316* |
| Severity = 1.6 mm impact depth (reference: 1.2 mm impact depth) | -0.0304 | 0.226 | -0.134 | 0.893 |

**Additional file: Table S5**: Output of multiple linear regression examining the effects of sex and impact severity on white matter damage in the optic tract. * = p < 0.05, ** = p < 0.01, *** = p < 0.001.

| Coefficient | β estimate | Standard error | t value | Pr(>\|t\|) |
| --- | --- | --- | --- | --- |
| Intercept | 0.637 | 1.04 | 0.615 | 0.541 |
| Number of injuries = 2x (reference: 1x) | 3.21 | 0.815 | 3.93 | 0.000180*** |
| Inter injury interval = 1 week (reference: 1 day) | 0.462 | 1.00 | 0.460 | 0.647 |
| Inter injury interval = 2 weeks (reference: 1 day) | -0.411 | 0.992 | -0.414 | 0.680 |
| Sex = female (reference: male) | -1.05 | 0.816 | -1.28 | 0.203 |
| Severity = 1.6 mm impact depth (reference: 1.2 mm impact depth | 2.53 | 0.817 | 3.10 | 0.00270** |
